# Impact of an Antimicrobial Stewardship program on mortality and consumption of antibiotics in the intensive care units of a pediatric referral hospital in Peru

**DOI:** 10.1017/ash.2025.10174

**Published:** 2025-10-06

**Authors:** Irma Carolina Fonseca-Rivera, Alejandra Pando-Caciano, Ysela Dominga Agüero-Palacios

**Affiliations:** 1 Unidad de Epidemiología, Red de Salud Leoncio Prado, Huánuco 10131, Perú; 2 Sub Unidad de Investigación e Innovación Tecnológica, Instituto Nacional de Salud del Niño San Borja, Lima 15037, Perú; 3 Department of Cellular and Molecular Sciences, School of Science and Philosophy, Universidad Peruana Cayetano Heredia, Lima 15102, Perú; 4 Departamento of de Estadística, Facultad de Ciencias Matemáticas, Universidad Nacional Mayor de San Marcos, Lima 15081, Perú

## Abstract

**Objective:**

This study aimed to evaluate the impact of an Antimicrobial Stewardship Program (ASP) on antibiotic consumption and mortality rates in the intensive care units (ICUs) of a national pediatric referral hospital in Lima, Peru.

**Materials and methods:**

The study population included patients under 18 years of age admitted to five ICUs during two distinct periods: prior to the implementation of the ASP (2016–2017) and following its implementation (2017–2018). Days of therapy (DOT) per 1,000 patient days were calculated to evaluate antibiotic consumption, and the results were compared between the two periods. Mortality rates and length of stay (LOS) were also assessed and compared. Differences in antibiotic consumption, mortality, and LOS were analyzed using χ2 and t tests.

**Results:**

Following ASP implementation, average antibiotic consumption decreased significantly from 750 DOT to 660 DOT (*p* = 0.01), indicating a 12% reduction. Notable declines in antibiotic use were observed specifically in the cardiology and burns ICUs, as well as in the subgroups of carbapenems, glycopeptides, and oxazolidinones. However, the analysis revealed no significant reduction in mortality rates or LOS following the implementation of the ASP.

**Conclusions:**

Our findings suggest that the implementation of an ASP in pediatric ICUs is associated with a reduction in antibiotic consumption. These results underscore the critical importance of ASPs in promoting appropriate and rational use of antibiotics. Further research should explore the complex relationship between inappropriate antibiotic use and mortality in pediatric ICUs to clarify how ASP implementation may contribute to reducing mortality in this vulnerable population.

## Introduction

Antibiotics are frequently prescribed in pediatric intensive care units (ICUs) due to the increased risk of infections among these patients. In Latin America, the mortality rate in pediatric ICUs is significantly higher than that in European pediatric ICUs.^1^ Furthermore, antibiotic consumption in pediatric ICUs in Latin America is approximately 3.5 times greater.^2^ A substantial proportion of antibiotic prescriptions in ICUs are made empirically (33%) and inappropriately (34%), which contributes to the escalating problem of antimicrobial resistance.^3^ Rates of ICU-acquired infections in hospitals of developing countries can reach as high as 83%, with infections caused by multidrug-resistant and extensively resistant organisms affecting 61% and 31% of patients, respectively.^4^ Under these circumstances, patient survival largely depends on the effectiveness of the administered antibiotics and their appropriate use. In that sense, the World Health Organization has been advocating for the implementation of strategies to optimize antimicrobial stewardship in both adult and pediatric clinical settings.^5^

Antimicrobial stewardship programs (ASPs) have been identified as effective strategies to promote the appropriate use of antibiotics, with the primary aim of mitigating the rise of bacterial resistance while optimizing clinical outcomes and reducing healthcare costs.^6^ The implementation of ASPs in pediatric hospitals has been shown to lead to a significant decrease in antimicrobial consumption and rates of antimicrobial resistance, while also enhancing the rate of appropriate prescribing.^7,8^ Furthermore, a reduction in mortality rates has been observed following the introduction of a carbapenem-focused ASP, underscoring the potential of these interventions to improve patient clinical outcomes.^9^

Previous studies on antibiotic consumption in pediatric patients have primarily focused on evaluating the reduction in the use of these medications within general hospital services. However, information regarding antibiotic consumption in pediatric ICUs and its association with mortality remains limited, particularly in Latin America.^10,11^

Considering the existing evidence on the impact of ASPs in reducing inappropriate antibiotic consumption, the Instituto Nacional de Salud del Niño San Borja (INSN-SB), a pediatric referral center in Lima, Peru, launched an ASP in 2017 for implementation in the hospital’s ICUs. This ASP was designed based on two primary strategies: restricting antibiotic dispensing and implementing prospective audits to assess the appropriateness of antimicrobial prescriptions. This initiative led to a significant reduction in expenditures on antibiotics, reaching approximately 2.5 million soles within the first six months following its implementation.^12^

Despite the concerning rise in the resistance rates of g microorganisms in Latin America, the introduction and subsequent evaluation of ASPs in pediatric clinical settings remain limited across the region.^13^ In this context, the present study aimed to assess the impact of ASP implementation on antibiotic consumption and mortality rates in five ICUs of the INSN-SB. This was accomplished through a comparative analysis of data from the periods preceding (2016–2017) and following (2017–2018) the implementation of the ASP.

## Materials and methods

### Study design and population

This study employed a retrospective observational analytical design. Mortality rates and antibiotic consumption were compared between two distinct periods: the preimplementation phase of the ASP (June 2016 – May 2017) and the postimplementation phase (June 2017 – May 2018).

The study population included all patients admitted to the five ICU wards at the INSN-SB, which comprise the cardiology ICU, cardiovascular ICU, neonatology ICU, neurosurgery ICU, and burns ICU. Inclusion criteria permitted only those patients who remained in the ICU for a minimum of 72 hours and received at least one antibiotic during their hospitalization, irrespective of whether the antibiotic was prescribed with or without microbiological support.

### Implementation of the ASP

In 2017, the INSN-SB implemented an ASP titled “Annual Plan for Monitoring and Control of the Use of Restricted Antimicrobials in Intensive Care Units.” For the purposes of this study, the term ASP will be used to refer specifically to this initiative. The ASP encompassed a broad range of restricted, broad-spectrum antimicrobials, including antifungals, antivirals, and antibacterials. However, this analysis focused exclusively on systemic antibacterials.

This ASP was based on two primary strategies aimed at regulating antibiotic use: (1) The restriction of antibiotic dispensing, which required approval from an infectious disease specialist and adherence to the guidelines outlined in the “Directive for the Use of Restricted Antimicrobials in the INSN-SB” ^14^ and (2) The conduct of prospective audits, wherein the appropriateness of antimicrobial prescriptions was evaluated by a pharmaceutical chemist and an infectious disease specialist. Factors such as correct dosage, potential drug-drug interactions, and options for de-escalation were considered during these evaluations. The findings were subsequently presented to the Healthcare Infection Control Practices Advisory Committee and the Technical Coordination of Epidemiology, who jointly determined the administration of the antimicrobial treatment.

### Procedures

Sociodemographic and clinical data were extracted from the electronic medical records. The variables collected included age, medical record ID, number of ICU admissions, sex, presence of comorbidities, ICU admission and discharge dates, and hospital discharge status (deceased or alive). The data were organized according to the medical record ID.

Furthermore, information regarding the names and pharmacological formulations of the antibiotics administered to the patients, including the quantities dispensed (measured in units) and the dates of prescription, was collected.

### Data analysis

Antibiotics were classified into chemical subgroups according to the Anatomical, Therapeutic, and Chemical Classification System (ATC). Antibiotic consumption was quantified by calculating the Days of Therapy (DOT). DOT were reported as DOT per 1,000 patient days for each period and ICU. In cases where patients received combination antibiotic therapy, DOT for each chemical subgroup were aggregated. Variables were summarized using frequencies and percentages, or means and standard deviations (SD). Differences between groups were evaluated using χ^2^ tests and T-tests. Statistical significance was considered at a *P–* value of < 0.05.

The hospitalwide mortality rate was determined by dividing the total number of deaths by the overall number of admissions to ICUs. Furthermore, the individual mortality rates for each ICU were calculated. For the purposes of this study, only deaths that occurred more than 72 hours after hospitalization were included in the analysis.

Data analysis was performed using the Statistical Package for the Social Sciences (SPSS) version 26.

### Ethical aspects

This research was approved by the Institutional Ethics Committee of the INSN-SB (code PI-487). The antibiotic consumption database was provided by the Department of Statistics without any personal identifiers; only unique ID records were maintained. Each participant was assigned a distinct numerical code to ensure anonymity prior to data analysis. Additionally, to protect data confidentiality, access to the project database was restricted to authorized personnel only, safeguarded by access controls. Given the retrospective nature of the study, informed consent was not required.

## Results

Sixteen unique antibiotic types were administered to the patients in this study. As detailed in Table 1, these antibiotics were classified into twelve distinct chemical subgroups.

**Table 1.** Chemical subgroups of the antibiotics used by the patients included in the study

ATC Code (Level 4)	Chemical subgroup	Antibiotic
J01CA	Broad-spectrum penicillins	Ampicillin
J01CR	β-Lactam antibiotics	Ampicillin + Sulbactam
		Piperacillin + Tazobactam
J01DD	Third-generation cephalosporins	Cefotaxime
		Ceftazidime
		Ceftriaxone
J01DE	Fourth-generation cephalosporins	Cefepime
J01DH	Carbapenems	Meropenem
		Imipenem + Cilastatin
J01EE	*S*ulfonamide and trimethoprim	Sulfamethoxazole + Trimethoprim
J01GB	Other aminoglycosides	Amikacin
J01MA	Fluoroquinolones	Ciprofloxacin
J01XA	Glycopeptides	Vancomycin
J01XB	Polymyxins	Colistin
J01XD	Imidazole derivatives	Metronidazole
J01XX	Oxazolidinones	Linezolid

During the study period, a total of 2,184 ICU admissions were recorded. Among these, 1,655 admissions met the inclusion criteria and were included in the analysis. The preASP period accounted for 749 admissions involving 611 patients, while the postASP period comprised 906 admissions involving 727 patients. The clinical and epidemiological characteristics of the patients included in the study are detailed in Table 2. With the exception of age, no statistically significant differences were noted between the two periods regarding sex, discharge status, the ICU where patients were admitted, antibiotic consumption, or the presence of comorbidities.

**Table 2.** Sociodemographic and clinical features of the patients included in the study

Variable	PreASP (*n* = 749)	PostASP (*n* = 906)	*p*-value
ICU length of stay (Average number of days ± SD)	14.35 ±16.22	15.05 ±16.35	.39
Sex			
Female	312 (41.70%)	408 (45.00%)	.17
Male	437 (58.30%)	498 (55.00%)	
Age group (years)			
Under 1 year	438 (58.50%)	495 (54.60%)	.03
1 to 4 years	163 (21.80%)	242 (26.70%)	
5 to 12 years	118 (15.80%)	120 (13.20%)	
>12 years	30 (4.00%)	49 (5.40%)	
Discharge status			
Alive	669 (89.30%)	829 (91.50%)	.13
Deceased	80 (10.68%)	77 (8.50%)	
ICU			
Cardiovascular	246 (32.80%)	290 (32.00%)	.13
Neonatology	148 (19.80%)	164 (18.10%)	
Cardiology	93 (12.40%)	152 (16.80%)	
Neurosurgery	136 (18.20%)	166 (18.30%)	
Burns	126 (1.80%)	134 (14.80%)	
Antibiotic consumption			
Yes	625 (83.40%)	745 (82.20%)	.51
No	124 (16.60%)	161 (17.80%)	
Comorbidities			
Present	665 (88.80%)	785 (86.60%)	.19
Absent	84 (11.20%)	121 (13.40%)	

A total of 157 deaths were recorded throughout the study period. In the preASP implementation phase, 80 deaths were observed, accounting for 10.70% of the patient population. Conversely, in the postASP implementation phase, 77 deaths were documented, representing 8.50% of the population. Of the 80 patients who died during the preASP period, only 7 (5.6%) did not receive antibiotic treatment. In contrast, among the 77 patients who died during the postASP period, only 3 (1.9%) did not receive antibiotics.

In the preASP period, antibiotic use was reported in 625 admissions (83.40%). In contrast, antibiotic use was documented in 745 admissions (82.20%) during the postASP period. The total DOT recorded in the pre and postASP periods were 562.04 and 597.66, respectively. A statistically significant difference was observed in the average total antibiotic consumption, measured as DOT per 1,000 patient days, between the two periods across the five ICUs, with values of 750.60 in the preASP period compared to 660.53 in the postASP period (*P* = 0.01). This indicated a 12% reduction in antibiotic consumption following the implementation of the ASP.

The most frequently consumed antibiotic subgroups were carbapenems, followed by glycopeptides, other aminoglycosides, and third-generation cephalosporins, in that order. A comparison of overall consumption across these subgroups revealed a significant decrease in average usage between the preASP and postASP periods for carbapenems (*P* = 0.01), glycopeptides (*P* = 0.00), and oxazolidinones (*P* = 0.01) (Table 3). The most notable reduction was observed in the category of oxazolidinones (72.41%), followed by glycopeptides (22.76%) and carbapenems (18.92%).

**Table 3.** Overall consumption of antibiotics (DOT/1000 patient-days) in the pre and postASP periods, by chemical subgroup

Chemical subgroup	DOT/1000 p-d (Mean ± SD)		*p*-value
	PreASP	PostASP	
**β-Lactam antibiotics**	60.0 ± 150.8	59.2 ± 142.8	.91
**Third-generation cephalosporins**	111.1 ± 227.7	113.3 ± 232.5	.85
Carbapenems	177.6 ± 284.7	144.0 ± 249.0	.01
Other aminoglycosides	138.8 ± 236.6	140.6 ± 247.7	.88
**Broad-spectrum penicillins**	39.3 ± 138.8	33.7 ± 132.6	.40
**Fourth-generation cephalosporins**	10.3 ± 68.2	4.8 ± 52.2	.07
Fluoroquinolones	6.9 ± 60.0	2.9 ± 37.9	.11
Polymyxins	15.8± 86.4	9.9 ± 62.3	.12
Oxazolidinones	11.6 ± 84.4	3.2 ± 38.0	.01
Imidazole derivatives	15.6 ± 74.9	16 ± 93.9	.92
**Sulfonamide and trimethoprim**	5.4 ± 54.6	10 ± 71.2	.14
Glycopeptides	158.2 ± 261.5	122.2 ± 226.2	<.001

p-d: patient-days.

Similarly, when overall consumption was evaluated across different ICUs, a significant decrease was observed in the cardiology (*P* = 0.02) and burns (*P* = 0.01) units (Table 4).

**Table 4.** Overall consumption of antibiotics (DOT/1000 patient-days) in the pre and postASP periods, by ICU

ICU	PreASP	PostASP	*p*-value
	DOT/1000 p-d (Mean ± SD)	DOT/1000 p-d (Mean ± SD)	
Cardiovascular	549.90 ± 466.51	543.10 ± 411.72	.86
Neonatology	790.50 ± 495.97	716.30 ± 513.33	.20
Cardiology	864.10 ± 743.84	649.40 ± 500.13	.02
Neurosurgery	798.70 ± 632.17	748.70 ± 611.34	.49
Burns	957.70 ± 675.41	745.00 ± 665.98	.01

p-d: patient-days.

No significant differences were observed when comparing the hospitalwide mortality rate and the mortality rates across each ICU between the pre and postASP periods (Table 5). Additionally, there were no differences in mortality rates across the different age groups during these comparison periods (Table 6).

**Table 5.** Mortality rates in the pre and postASP periods, by ICU

ICU	PreASP		PostASP		*p*-value
	Deceased (*n*)	MR (%)	Deceased (*n*)	MR (%)	
Cardiovascular	26	10.57	24	8.28	.45
Neonatology	7	4.73	3	1.83	.26
Cardiology	21	22.58	34	22.37	1.00
Neurosurgery	16	11.76	13	7.83	.34
Burns	10	7.94	3	2.24	.07
Total	80	10.68	77	8.50	.15

MR: mortality rate.

**Table 6.** Mortality rates in the pre and postASP periods, by age group

Age group (years)	PreASP		PostASP		*p*-value
	Deceased (*n*)	MR (%)	Deceased (*n*)	MR (%)	
<1	56	12.79	51	10.30	.28
1–4	14	8.59	12	4.96	.21
5–12	8	6.78	11	9.17	.66
>12	2	6.67	3	6.12	1.00

MR: mortality rate.

The length of stay in the ICU ranged from 3 to 130 days during the preASP period and from 3 to 178 days in the postASP period (Table 7). The maximum length of stay in the ICU was 178 days during the preASP period, compared to 130 days in the postASP period. No significant differences were observed between the preASP and postASP periods with respect to this variable.

**Table 7.** Length of stay in ICU in the pre and postASP periods

Period	Length of stay (days)				*p*-value
	Minimum	Maximum	Average	DE	
PreASP	3	178	14.35	16.22	.39
PostASP	3	130	15.05	16.35	

## Discussion

The implementation of ASPs in pediatric clinical settings has demonstrated a significant reduction in antibiotic consumption, shorter hospital stays, and decreased mortality rates in ICUs. Additionally, ASPs have been associated with an increase in the appropriate prescribing of antibiotics.^7^ Notably, a substantial proportion of patients admitted to these units (42%) receive antibiotics empirically, often with more than three different antibiotics prescribed concurrently.^15^ Furthermore, the introduction of ASPs in pediatric ICUs has led to a remarkable decline in antibiotic resistance rates, with reductions from levels as high as 100% to less than 50% for certain antibiotics, including clindamycin, erythromycin, and tetracycline.^8^ Previously implemented ASPs in pediatric wards include telehealth-based approaches, software-supported programs, and administrative models based on the application of institutional guidelines and recommendations, as exemplified in this study.^16–18^

In the present study, we observed a significant reduction in antibiotic consumption in the ICUs following the implementation of an ASP. Additionally, our findings demonstrated that the absence of the ASP was not associated with an increased mortality risk or prolonged length of stay among this patient population, consistent with findings from other studies performed in pediatric ICUs.^17,19,20^ This represents the first investigation conducted in Peru assessing changes in antibiotic utilization and mortality rates following the implementation of an ASP in a pediatric hospital, as well as the potential association between these variables.

Consistent with the findings of this study, a notable reduction in antibiotic consumption, measured in DOT per 1,000 patient days, has been documented in previous research conducted within pediatric ICUs. However, unlike the 12% reduction observed in our study, earlier studies reported significantly higher reductions, ranging from 18% to 47%.^17,19,21,22^ The intervention resulting in the most substantial decrease in antibiotic consumption (47%) involved the implementation of an ASP using telemedicine. This approach was designed to address the shortage of specialists qualified to evaluate the appropriateness of antimicrobial prescriptions, assess prescribed dosages, verify the presence of pathogens and antimicrobial resistance, conduct therapeutic monitoring, and review potential drug interactions. Although a telemedicine-based ASP was not utilized in this study, it is important to recognize that adopting this approach in a developing country such as Peru presents various challenges, including dependence on local infrastructure and internet connectivity, as well as variability in access to technology and training among healthcare personnel.^23^

The modest reduction in antibiotic use observed in this study, compared to previous research, may be attributed to the short follow-up period, which could limit the ability to fully evaluate the true impact of the ASP when comparing the pre and postimplementation phases. Additionally, as the ASP underwent an adaptation phase during its initial months, challenges with protocol adherence among healthcare personnel may have impacted the outcomes compared to the preintervention year.

Although additional studies conducted in pediatric ICUs have shown a significant reduction in antibiotic consumption following the implementation of an ASP, a comparative analysis of antibiotic consumption before and after the program’s introduction has not been conducted.^18,20,24,25^ One successful ASP involved the use of software to monitor patients prescribed antimicrobials, enabling alerts when the administration time indicated by the physician was exceeded.^18^ This facilitated timely evaluation of whether to continue or discontinue the medication. Another effective intervention aimed at reducing antimicrobial consumption, particularly in neonates, involved the early discontinuation of antibiotics in patients lacking evidence of infection risk factors.^24^ This included neonates who presented without clinical sepsis, had a C-reactive protein value ≤ 10 mg/L during the first 72 hours of life, and displayed negative cultures within the first three days of antibiotic therapy. Collectively, these findings underscore the efficacy of ASPs in minimizing antibiotic consumption within pediatric ICUs.

In this study, the most frequently used antibiotic subgroups were identified as carbapenems, followed by glycopeptides, other aminoglycosides, and third-generation cephalosporins, in that order. This finding aligns with reports from pediatric ICUs in Brazil, where the highest antibiotic consumption was attributed to glycopeptides—specifically vancomycin and dalbavancin—with an average of 220.95 DOT per 1,000 patient days.^2^ This was followed by macrolides, which included erythromycin, clarithromycin, and azithromycin, accounting for 163.5 DOT per 1,000 patient days. In contrast, data from pediatric ICUs in Saudi Arabia indicated that third-generation cephalosporins were the most consumed antibiotics, with ceftriaxone leading at 164.8 DOT per 1,000 patient days, followed by ceftazidime at 91.7 DOT and cefotaxime at 42.9 DOT. Glycopeptides consumption was also significant, with vancomycin reaching a rate of 150.5 DOT per 1,000 patient days.^26,27^

Variations in regional antibiotic prescription patterns may be attributed to differences in baseline consumption. A systematic review documented that implementation of ASPs was associated with an average 6% reduction in antibiotic prescribing in high-income countries (HICs), compared with a 30% reduction in low- and middle-income countries (LMICs), suggesting that baseline consumption is generally lower in HICs than in LMICs. These findings underscore the importance of developing tailored stewardship interventions that consider local antibiotic consumption profiles.^28^

The current study observed a reduction in antibiotic consumption, specifically among drugs belonging to the chemical subgroups of carbapenems, oxazolidinones (linezolid), and glycopeptides. Notably, the most significant reduction was recorded for the oxazolidinone group, with a decrease of 72.41%. This finding is particularly noteworthy as reductions in the consumption of these antimicrobials have not been reported in previous studies. In contrast, prior research has documented declines in the consumption of carbapenems and glycopeptides following the implementation of ASPs in pediatric ICUs, aligning with the results of the present study.

For instance, Alfraij *et al*. and Bassiouny *et al*. documented reductions in carbapenem use (imipenem and meropenem) of 73.41% and 70.92%, respectively, in hospitals located in Kuwait and Egypt.^17,20^ Bassiouny *et al*. also observed a decrease in glycopeptides consumption, particularly vancomycin, of 64.04%. Similarly, Renk *et al*. documented a reduction of 57% in glycopeptides use in hospitals in Germany.^20,21^ In the present study, reductions in carbapenems and glycopeptides reached approximately 18.92% and 22.76%. These findings reflect reductions roughly four times and three times smaller than those reported in the aforementioned studies.

In Latin America and Spanish-speaking countries, research examining the impact of ASPs on antibiotic consumption is limited, with many studies lacking standardized measures for reporting consumption, such as DOT. A study conducted at a tertiary hospital in Panama reported a reduction in the consumption of gentamicin, vancomycin, meropenem, cefotaxime, and ceftazidime, assessed by the number of vials dispensed annually, during the postintervention period compared to the preintervention period. This finding aligns with the results of the present study regarding the decreased consumption of carbapenems (specifically meropenem) and glycopeptides (vancomycin).^29^ Another study conducted in a tertiary pediatric hospital in Spain documented an overall reduction of 27.8% in DOT per 1,000 hospital admissions and a decrease of 22.9% in the number of prescriptions for each antibiotic following the implementation of an ASP, although these differences did not achieve statistical significance. Similarly, our investigation revealed a decline in the specific use of carbapenems (meropenem and imipenem/cilastatin), glycopeptides (vancomycin), and oxazolidinones (linezolid).^30^ Reducing the use of carbapenems, glycopeptides, and oxazolidinones is particularly important because these agents are often considered last-resort antibiotics for treating multidrug-resistant infections. Limiting their use may help prevent the development and spread of antimicrobial resistance, thereby maintaining their effectiveness for severe infections.

Inappropriate antibiotic prescriptions have been reported to be associated with higher mortality rates in pediatric ICUs, with rates as high as 33.8%.^31^ Given the significant difference in mortality rates reported in pediatric ICUs in Latin America and Europe (13% vs 5%, *P* = 0.005), an evaluation of the impact of ASPs on mortality reduction in these settings became necessary. In this study, no decrease in mortality was documented following the implementation of an ASP in pediatric ICUs. These findings align with previous investigations conducted by the same research group, as well as other studies in similar environments, all reporting no significant reduction in mortality associated with ASP implementation.^16,17,19–22,25,32,33^ Conversely, research conducted in developing countries indicates a decrease in hospital mortality rates ranging from 23% to 28% following ASP implementation.^34,35^ The absence of a significant impact on mortality rates after ASP implementation suggests that mortality may be influenced not only by inappropriate antibiotic prescribing practices but also by patient-related factors, including fluid overload greater than 10%, low birth weight (< 1.8 kg), comorbidities such as anemia or gastrointestinal hemorrhage, and severe pneumonia; therapeutic interventions, such as administration of sedative-hypnotics; and clinical procedures, including mechanical ventilation.^36,37^

An additional indicator assessed to evaluate the impact of implementing an ASP on clinical outcomes was the length of stay in the ICU. Several prior studies have shown no significant reduction in length of stay following ASP implementation, which aligns with the findings of this study.^16,17,19,21,22,32,38^ However, two investigations conducted in neonatal ICUs in Egypt and Greece reported a significant decrease in ICU length of stay after ASP application. In the Egyptian NICU, the average length of stay decreased from 15.15 days to 11.94 days (*P* = 0.027), while the Greek ICU experienced a reduction of 2 days (*P* = 0.043).^20,24^ Similar to mortality, the length of stay in pediatric ICUs is influenced by factors beyond antibiotic prescribing practice. These determinants include patient-specific characteristics such as central nervous system comorbidities; clinical procedures including tracheostomy, peripherally inserted central venous catheterization, and mechanical ventilation; and hospital-acquired infections like pneumonia, bloodstream infections, and urinary tract infections.^39^ In addition, differences in patient age between the preASP and postASP periods may have acted as a minor confounder in the association between antibiotic consumption and clinical outcomes (mortality and length of stay).

This research has inherent limitations due to its retrospective design, which may have led to incomplete data from the sources employed. To address this limitation, we included all patients for whom relevant variables were available. However, we recommend that future studies adopt a prospective approach to incorporate additional variables that could further clarify the relationship between inappropriate antibiotic use and mortality in the ICU. These variables may be included in a multivariate logistic regression analysis as potential confounders to provide a comprehensive view of this relationship. According to previous studies, key factors warranting attention include the presence of comorbidities and infections, the application of clinical procedures, and the treatments administered.^36–38^ We further hypothesize that additional variables, such as the class and specific dosages of antibiotics administered, the duration of therapy, the underlying causes of death, as well as the species of microorganisms identified in cultured samples, along with their resistance profiles, may also influence mortality in pediatric ICUs. Moreover, future research should examine the potential effects of antibiotic consumption on cost reduction and antimicrobial resistance rates within pediatric ICUs, as demonstrated in previous studies.^17,18,21^

A significant limitation in studies reporting antibiotic consumption lies in the lack of standardization in the units of measurement utilized. Typically, antibiotic consumption is expressed in terms of DOT or defined daily doses (DDD). The calculation of DDD relies on dosages assigned by the World Health Organization for adults, which can differ from those commonly used in clinical practice, particularly in pediatric settings where dosing recommendations vary according to age and body weight. Thus, using DOT instead of DDD is advisable to avoid overestimating antimicrobial consumption.^40,41^ DOT offers the advantage of independence from both the number of doses administered and the specific dosages used, making it the preferred metric for reporting antibiotic consumption in this context. Additionally, previous studies on antibiotic consumption in pediatric ICUs have documented other less frequently used measures, including the proportion of use—defined as the number of patients receiving antibiotics divided by the total number of patients included in the study—and the number of doses dispensed per bed-day.^7,42^

## Conclusions

The findings of this study provide robust evidence demonstrating that the implementation of an ASP in the INSN-SB ICUs was associated with a significant reduction in antibiotic consumption. However, the analysis revealed no significant change in mortality rates or length of stay across the five ICUs. These results emphasize the vital importance of ASPs in fostering the appropriate and rational use of antibiotics. To further clarify the impact of ASP implementation on mortality rates, additional prospective studies should explore the complex relationship between inappropriate antibiotic use and mortality in pediatric ICUs. Such investigations should take into account factors related to patient clinical status, the nature of infections (including microorganism types and resistance profiles), the treatments administered for other medical conditions beyond the infection, and other relevant variables to provide a more comprehensive understanding of this relationship.

## Research transparency and reproducibility

The datasets generated during the current study are available from the corresponding author upon reasonable request.
