# Impact of an Antimicrobial Stewardship program on mortality and consumption of antibiotics in the intensive care units of a pediatric referral hospital in Peru – ADDENDUM

**DOI:** 10.1017/ash.2025.10236

**Published:** 2025-12-12

**Authors:** Irma Carolina Fonseca-Rivera, Alejandra Pando-Caciano, Ysela Dominga Agüero-Palacios

In the original publication of this article^1^, one of the affiliations of author Irma Carolina Fonseca-Rivera was omitted due to an error that occurred during production. The article has been updated to include this affiliation: Facultad de Ciencias de la Salud, Universidad de Huánuco, Huánuco 10001, Perú.
